# Draft genome sequence of *Thermoactinomyces sp*. Gus2-1 isolated from the hot-spring Gusikha in Bargusin Valley (Baikal Rift Zone, Russia)

**DOI:** 10.1016/j.gdata.2016.11.014

**Published:** 2016-11-14

**Authors:** Aleksey S. Rozanov, Alla V. Bryanskaya, Anastasia V. Kotenko, Sergey E. Peltek

**Affiliations:** Federal Research Center “Institute of Cytology and Genetics of the Siberian Branch of the RAS”, Novosibirsk, Russia

## Abstract

The *Thermoactinomyces* sp. strain Gus2-1 was isolated from hot-spring sediments sample from the hot-spring Gusikha in Bargusin Valley (Baikal Rift Zone, Russia). The sequenced and annotated genome is 2,623,309 bp and encodes 2513 genes. The draft genome sequence of the *Thermoactinomyces* sp. strain Gus2-1 has been deposited at DDBJ/EMBL/GenBank under the accession JPZM01000000 and the sequences could be found at the site https://www.ncbi.nlm.nih.gov/nuccore/JPZM01000000.

**Table:** 

Specifications	
Organism/cell line/tissue	*Thermoactinomyces sp*. Gus2–1
Sex	–
Sequencer or array type	Ion PGM™ Template OT2 400 kits
Data format	Processed
Experimental factors	Bacteria
Experimental features	Whole genome sequence of *N. lepida*, assembly and annotation
Consent	Level of consent allowed for reuse if applicable
Sample source location	The hot-spring Gusikha (60 °C) in Bargusin Valley (Baikal Rift Zone, Russia)

## 1 Direct link to deposited data

https://www.ncbi.nlm.nih.gov/nuccore/JPZM01000000.

## 2 Introduction

The genus *Thermoactinomyces* is one of the earliest known Actinomycete taxa, and the type species of this genus is *Thermoactinomyces vulgaris* [1]. The members of this genus are aerobic, endospore-forming, Gram-positive bacteria belonging to the order *Bacillales* [2]. They produce endospores that are formed endogenously inside the aerial and substrate hyphae of the bacteria [3]. Currently, researchers are finding new species belonging to this genus [4].

## 3 Strain isolation

The strain Gus2-1 was isolated from sediments sample from the hot-spring Gusikha (60 °C) in Bargusin Valley (Baikal Rift Zone, Russia). *Thermoactinomyces sp.* Gus2-1 culture was cultivated in liquid medium containing 1% trypton, 0.5% yeast extract, and 3.5 M of NaCl. Eight ml of cell culture were pelleted by centrifugation and resuspended in 75 μl of H_2_O by intense pipetting.

## 4 DNA isolation and sequencing

DNA was isolated using the DNA Purification Kit (Fermentas). Ion PGM™ Template OT2 400 and Ion PGM™ Template OT2 400 kits were used to create libraries for genome sequencing. Genome sequencing was performed on an IonTorrent platform (Applied Biosistems) using Ion PGM™ Sequencing 400 Kit in the SBRAS Sequencing Center.

## 5 Genome assembly and annotation

*De novo* assembly of short reads into contigs was performed using MIRA v. 4. Contigs shorter than 1000 bp were deleted. A total of 92 contigs yielded a genome sequence 2,623,309 bp long, and the G + C content is 48.01%. ORF prediction and automatic annotation was performed using NCBI PGAAP (http://www.ncbi.nlm.nih.gov/genome/annotation_prok). The complete genome sequence contained 2513 genes, 2315 CDS, 14 rRNAs (5S, 16S, 23S), 76 tRNAs, one ncRNA.

## 6 Phylogenetic analysis

Phylogenetic analysis was performed using 16S rRNA sequences with the UPGMA algorithm implemented in MEGA v.6. 16S rRNA sequences of *Thermoactinomyces* type strains were found using the StrainInfo (www.straininfo.net) and GenBank (www.ncbi.nlm.nih.gov/nucleotide) databases. According to phylogenetic analysis, the *Thermoactinomyces* sp. strain Gus2-1 is most closely related to *Thermoactinomyces vulgaris*.

## 7 Nucleotide sequence accession numbers

The draft genome sequence for *Thermoactinomyces* sp. strain Gus2-1 has been deposited in DDBJ/EMBL/Genbank under the accession no. JPZM01000000. The 92 contigs have been deposited under accession no. JPZM01000001-JPZM01000092.

## Conflict of interest

The authors declare that there is no conflict of interests with respect to the work published in this paper.
